# Genome Organization in and around the Nucleolus

**DOI:** 10.3390/cells8060579

**Published:** 2019-06-12

**Authors:** Cristiana Bersaglieri, Raffaella Santoro

**Affiliations:** 1 Department of Molecular Mechanisms of Disease, DMMD, University of Zurich, 8057 Zurich, Switzerland; cristiana.bersaglieri@dmmd.uzh.ch; 2 Molecular Life Science Program, Life Science Zurich Graduate School, University of Zurich, 8057 Zurich, Switzerland

**Keywords:** Nucleolus, rRNA genes, NoRC, chromatin, epigenetics, lncRNA, nucleolus-associated domains (NADs), lamina-associated domains(LADs), genome architecture, genome instability, embryonic stem cells, cancer

## Abstract

The nucleolus is the largest substructure in the nucleus, where ribosome biogenesis takes place, and forms around the nucleolar organizer regions (NORs) that comprise ribosomal RNA (rRNA) genes. Each cell contains hundreds of rRNA genes, which are organized in three distinct chromatin and transcriptional states—silent, inactive and active. Increasing evidence indicates that the role of the nucleolus and rRNA genes goes beyond the control of ribosome biogenesis. Recent results highlighted the nucleolus as a compartment for the location and regulation of repressive genomic domains and, together with the nuclear lamina, represents the hub for the organization of the inactive heterochromatin. In this review, we aim to describe the crosstalk between the nucleolus and the rest of the genome and how distinct rRNA gene chromatin states affect nucleolus structure and are implicated in genome stability, genome architecture, and cell fate decision.

## 1. Introduction

The nucleolus is the largest substructure in the nucleus, where ribosome biogenesis takes place, a process that is responsible for the assembly of the translational machinery, the ribosome, and is tightly regulated according to cell state. Ribosome biogenesis is initiated by the transcription of ribosomal RNA (rRNA) genes, the genetic component of the nucleolus. In eukaryotic genomes, rRNA genes are generally present in high copy numbers and arranged in arrays of tandem repeats among different chromosomes at regions called nucleolar organizer regions (NORs). rRNA genes play a crucial role in ribosome biogenesis, a highly coordinated process regulated by a myriad of factors. rRNA gene transcription generates 45S/47S pre-rRNA that is then modified and processed to form 28S, 18S and 5.8S rRNAs. These rRNAs are assembled with ribosomal proteins and 5S rRNA and exported from the nucleus to give rise to active ribosomes in the cytoplasm [1]. 

Recent studies indicate that the function of the nucleolus and the chromatin composition of rRNA genes go beyond the ribosome biogenesis. In this review, we aim to describe the different classes of rRNA genes, with a particular focus on the molecular features and the regulation of silent rRNA repeats and the crosstalk between the nucleolus and rRNA genes with the rest of the cell nucleus. We would like to highlight the pivotal role of the nucleolus in the regulation of genome stability and architecture, focusing on the evidence that the chromatin state of rRNA genes plays important roles in the regulation of genome organization and cell state.

## 2. rRNA Genes and Nucleolus 

The nucleolus is a membrane-less compartment that consists of the fibrillar center (FC), the dense fibrillar component (DFC), and the granular component (GC). Transcription of rRNA is thought to occur at the boundary between FC and DFC whereas processing of the pre-rRNA occurs in the DFC region and pre-ribosomal subunit assembly takes place in the GC region [2,3]. Recent results revealed that these nucleolar subcompartments represent distinct, coexisting liquid phases and that phase separation induces the formation of multilayered liquids that may facilitate sequential RNA processing reactions in a variety of ribonucleoprotein bodies [4]. The size of the nucleolus positively correlates with rRNA synthesis [2]. Dividing cells produced elevated amounts of rRNA and often possess large nucleoli whereas downregulation of rRNA gene transcription leads to a reduction in nucleolar size [5]. Elevated rRNA transcription and ribosome biogenesis is a common feature of many cancers [6]. Accordingly, tumor cells generally showed an increased size and/or number of nucleoli that is used by pathologists as a diagnostic marker for certain cancers [7].

Because of the large demand of ribosomes for protein synthesis, rRNA is the most abundant transcript in the cell. rRNA represents approximately 80% of the total RNA in a yeast cell and in proliferating mammalian cells. To meet the enormous ribosome biosynthetic demand, cells evolved a unique transcription system by using an efficient RNA polymerase machinery and by amplifying the number of rRNA genes to hundreds or even thousands of copies per genome. 

rRNA genes are transcribed by RNA Polymerase I (Pol I), a process that also requires at least two other basal factors, the upstream binding factor UBF and the TBP-TAFI complex SL1 also known as TIF1B [8,9]. Transcription of rRNA genes is very efficient as evidenced by the high density of Pol I on rRNA genes from yeast (about 50 polymerases/gene), CHO cells (114 polymerases/gene), and rat liver cells (101 polymerases/gene) that correspond to one polymerase every 132, 123, and 139 nucleotides, respectively [10,11,12]. Thus, the presence of many rRNA genes that transcribe at such high rates is clearly contributing to generate high levels of rRNA. 

The number of rRNA gene repeats varies enormously throughout phylogeny with humans and mice bearing ~200 rRNA genes per haploid genome, while amphibians and plants may have thousands of copies [13]. rRNA gene number can also vary between individuals of the same species or even between the cells of an individual [14]. In humans and apes, rRNA genes are located between the short arm and the satellite body of acrocentric chromosomes 13, 14, 15, 21, and 22 [15]. Standard laboratory strains of mice, which are thought to have originated mainly from a European subspecies, *Mus musculus domesticus*, and partially from an Asian subspecies, *M.m.musculus/molossinus*, have rDNA clusters within the centromeric regions of chromosomes 12, 15, 16, 18 and 19 [16,17]. Chromosomal regions containing rRNA genes are called nucleolar organizer regions (NORs). In human cells, NORs range in size from 50 kb to >6 Mb [18]. *Saccharomyces cerevisiae* contains 150–200 rRNA genes that are located on the right arm of chromosome XII, and cover about 60% of chromosome XII and about 10% of the whole genome [19,20]. The plant *Arabidopsis thaliana* has NORs on chromosomes 2 and 4, each consisting of ca. 375 rRNA genes and spanning 4 Mb [21].

## 3. A History of Silent, Inactive and Active rRNA Genes

**Figure 1:**
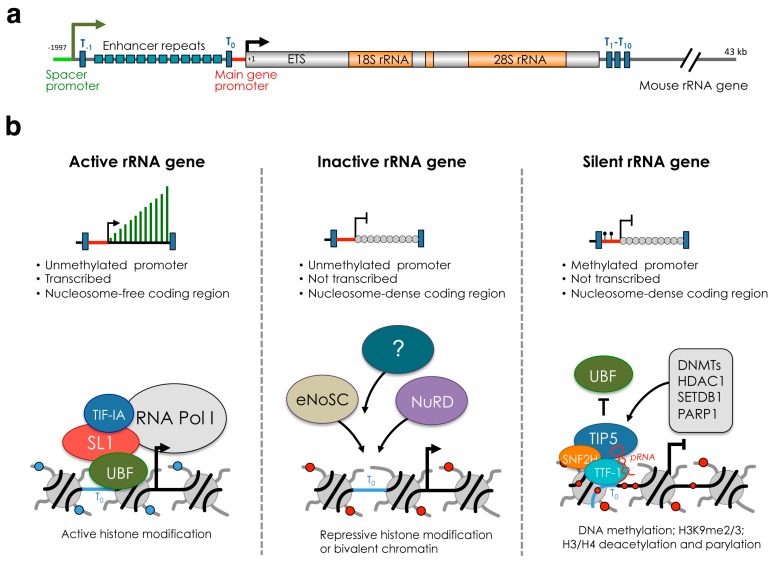
The three major classes of rRNA genes. (**a**) Structural organization of mouse rRNA gene. The sites of transcription initiation of the 45S pre-rRNA from the main gene promoter and IGS-rRNA transcripts from the spacer promoter are indicated by arrows. Terminator elements downstream of the spacer promoter (T-1), upstream of the main gene promoter (T0), and downstream of the coding regions (T1-T10) are marked by blue bars. The repeats composing the enhancer (13 according to the sequence from Genbank accession number BK000964) are shown. ETS, external transcribed spacer. (**b**) Description of active, inactive and silent rRNA genes based on transcription, chromatin and epigenetic state and factors regulating their state. The binding of UBF and NoRC (TIP5 and SNF2H) define active and silent rRNA genes. Inactive rRNA genes are non-transcribed repeats that lack promoter DNA methylation, are nucleosome-packed at the coding region, and are not bound by UBF or NoRC. The structure of inactive genes can be mediated by the nucleosome remodeling and deacetylase (NuRD) complex, the energy-dependent nucleolar silencing complex (eNOSC) or other yet unknown regulators.

Despite the high levels of rRNA gene transcription and the presence of many rRNA genes, not all rRNA genes within a cell are competent for transcription [22]. In mammalian cells, rRNA genes can be subdivided in three major classes according to the transcriptional state and chromatin and epigenetic features: silent, inactive (or pseudogenes), and active genes (Figure 1). In somatic cells, the presence of DNA methylation at the promoter region distinguishes silent rRNA genes from the rest of the repeats [23] (Figure 1b). Silent rRNA genes display heterochromatic structures and associate with repressive histone marks such as H3K9me2, H3K9me3 and deacetylated histones [24,25]. Similar features have also been observed in plant cells [26]. Furthermore, psoralen crosslinking experiments indicated that silent rRNA genes belong to the class of the non-transcribing and nucleosome-packed rDNA chromatin fraction [27]. In mammalian cells, silent rRNA genes replicate in mid-late S-phase, the time when heterochromatic DNA is usually duplicated and are inherited during cell division [28]. The presence of CpG methylation at the rRNA gene promoter abrogates the formation of the Pol I pre-initiation complex. Methylation of CpG at position −133 of the mouse rDNA promoter impairs the binding of UBF [23]. Accordingly, in mouse or human cells, UBF does not associate with the promoter of silent rRNA genes [24,29]. In the plant *Arabidopsis thaliana*, 50% of rRNA genes in somatic cells are silenced through epigenetic mechanisms that include DNA methylation and repressive histone modifications [30,31].

In mammalian cells, the rest of rRNA genes that do not belong to the silent fraction can be further subdivided into two groups, active and inactive genes (Figure 1b). These repeats do not contain DNA methylation, indicating that their transcription state can be potentially reversed. The key factor implicated in the establishment of active and inactive rRNA genes is UBF [29,32,33]. Active genes associate with UBF and are nucleosome-free in the coding region. UBF associates with the rRNA gene body, spacer promoter and enhancer repeats, allowing the formation of the pre-initiation complex (PIC) with subsequent RNA Pol I loading and rRNA transcription [33]. In contrast, inactive genes do not interact with UBF and belong to the nucleosome-packed rDNA chromatin as in the case of silent rRNA genes. Depletion of UBF switches active genes into inactive genes by promoting histone H1-induced assembly of transcriptionally inactive chromatin. Importantly, recovery of UBF expression restores the active gene number indicating that the switch from active to inactive state can be a reversible process [29]. 

In yeast *S. cerevisiae*, psoralen crosslinking analyses revealed the presence of two major classes of rRNA genes based on their nucleosome density [34]. Since yeast lack DNA methylation, these rRNA gene types can be considered to represent the active and inactive rRNA genes. These results are also supported by electron microscopy visualization with Miller spreading method showing rRNA genes with high Pol I loading and nascent rRNA and rRNA repeats that do not associate with Pol I and do not transcribe [10]. Since the major difference between active and inactive rRNA genes in yeast is the nucleosomal occupancy, it remains still unclear whether inactive rRNA genes can be inherited during cell division. Interestingly, replication of active rRNA genes in yeast generates two newly replicated rRNA genes with coding regions regularly packaged into nucleosomes [35]. These results clearly indicated that the chromatin structure of active rRNA genes is not directly inherited at the replication fork, and the re-establishment of the active state at rRNA repeats is always a post-replicative process, involving the disruption of preformed nucleosomes. 

A challenging question in the field is how silent, inactive, and active rRNA genes are distributed and where they localize within the nucleolus. In *S. cerevisiae* all rRNA genes are located at Chromosome XII and psoralen crosslinking experiments revealed that active and inactive copies are rather randomly distributed along the ribosomal rRNA gene locus [36]. In mammalian cells, rRNA gene loci are present at distinct chromosomes and the distribution of the three classes is not yet clear. Data suggest that NORs are generally either constitutively silent or competent for transcription. In metaphase chromosomes, where rRNA genes do not transcribe, active rRNA genes can be visualized by the persistent binding of Pol I transcription factors (UBF, SL1, and TTF1) on the repeats that were active in the preceding interphase [37,38,39]. A characteristic of these NORs in many if not all animals and plants is their ability to be selectively stained with silver nitrate (AgNORs) [40,41]. It is generally considered that NORs not positive for silver staining and not bound by Pol I factors are silent NORs. Importantly, both active and silent NORs are situated within nucleoli, suggesting that the transcription competence of NORs is not sufficient for the localization in the nucleoli, but other players are apparently involved as well [42]. Furthermore, TIP5, the factor responsible for the formation of silent rRNA genes is localized within the nucleolus of mammalian differentiated cells [43]. In human NORs, distal junctions (DJs) sequences are positioned immediately adjacent to the rRNA gene array on the telomeric side in linear chromosomal DNA. Interestingly, DJs are always found localized within the peri-nucleolar heterochromatin, pointing to the existence of ‘NOR territories’ within the nucleolus [44]. Studies in several human cell lines showed that active and silent NORs are inherited from one cell generation to the other one [45,46]. Since silent rRNA genes are also inherited through cell division and are marked by CpG methylation, an epigenetic mark that is maintained after the passage of the replication fork, it is likely that silent rRNA genes are located at silent NORs [28]. Accordingly, in early blastocysts the replication of all NORs is highly synchronized and takes place in early S phase [47]. Upon differentiation and concomitant with the de novo methylation at rRNA gene promoter regions [48], one copy of each NOR becomes late-replicating and this multi-chromosomal allelic pattern is then maintained clonally in somatic cells [47]. However, the absence of silent rRNA genes at active NORs in mammalian cells is still based on correlations and direct experimental evidences are still lacking due to technical limitations to visualize silent copies within a single NOR. Remarkably, in plant *Arabidopsis thaliana* ecotype (strain) Col-0, it was shown that silent and active rRNA genes are not intermingled. All silent rRNA genes were mapped to the NOR on chromosome 2 whereas all active rRNA gene subtypes were found on the NOR of chromosome 4 [49]. Interestingly, analysis of purified nucleoli of *A. thaliana* showed that active rRNA genes are present within nucleoli whereas silent copies are excluded [50]. In mutants with disruption of rRNA gene silencing, this nucleoplasmic-nucleolar partitioning is abrogated, suggesting that rRNA genes occupy distinct but changeable nuclear territories according to their epigenetic state. In mammalian cells, however, it remains still elusive whether silent rRNA genes occupy specific nucleolar space.

The results described above showed the classification of different classes of rRNA genes (silent, inactive and active copies in mammalian cells, active and inactive in yeast) based on epigenetic and chromatin features. Early evidence, however, indicates that rRNA genes can also differ in their sequence due to the presence of polymorphysms in both human and mouse rRNA genes [51,52], opening the question whether changes in the sequence can affect transcription and chromatin states. Recently, a variation was shown in the promoter methylation at a class of rRNA genes with genetic variation at position –104 (C or A) in a mouse maternal protein restriction model [53]. CpG-133 methylation levels were substantially lower for the C-variant relative to the A-variant, suggesting that in utero nutritional deficits influence offspring growth through epigenetic states at multicopy ribosomal DNA elements. Mouse rRNA genes can also be classified according to a polymorphism located at +42/+43 (rDNA-A, rDNA-T and rDNA-G) [54]. In mouse NIH3T3 cells, rDNA-A genes lack DNA methylation and are active whereas about 70% of rDNA-T and 50% of rDNA-G genes are CpG-methylated (i.e. silent copies). However, this DNA methylation pattern seems to be a property of NIH3T3 cells and it was not found in other mouse cell lines or tissues (unpublished data). Mouse rRNA genes can also vary the number of enhancer repeats located between the spacer promoter and the promoter-proximal terminator T0 [55,56,57]. Data from mouse cell lines and tissues revealed that rRNA gene variants have identical spacer and promoter sequences but contain a variable number (6, 9, 10, 11, 12, and 22) of enhancer repeats [57]. Interestingly, transcription from the spacer promoter, which in mouse is located 2 kb upstream of the main gene promoter, occurs at rRNA genes containing 9 or 6 enhancer repeats, indicating that these repeats have specialized functions in the synthesis of intergenic spacer transcripts (IGS-rRNA). In the plant *Arabidopsis thaliana Col*-0 strain, four rRNA gene types have been identified based on differences within a repetitive region of the external transcribed spacer (ETS) located just 3′ of the 25S rRNA sequences [49]. The VAR1 rRNA gene class accounts for ∼50% of the total rRNA gene pool, is located at NOR2, and is always silent. However, when VAR1 genes where placed in the active NOR4 they became transcriptionally active, indicating that selective rRNA gene silencing is not regulated on gene sequence variation.

Together all these results provide a complex picture of the organization of rRNA genes in term of chromatin structure and position in the nucleolus. In the following chapters, we will focus on the regulation of silent rRNA genes. Detailed information about the structure and regulation of active and inactive genes can be found in recent reviews [9,58].

## 4. Establishment and Maintenance of Silent rRNA Genes

**Figure 2:**
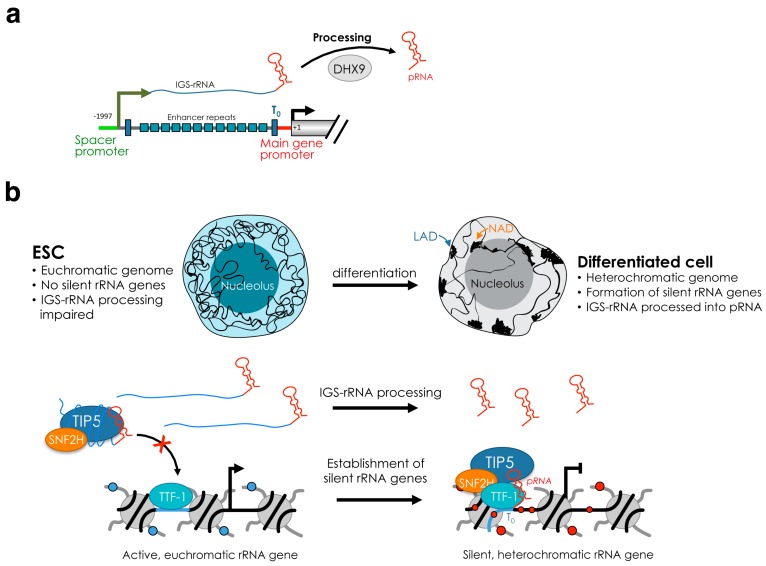
Regulation of rRNA gene chromatin state embryonic stem cells. (**a**) Scheme showing the synthesis of IGS-rRNA from the mouse spacer promoter and its processing into pRNA, a reaction mediated by DHX9 [57,64]. (**b**) Model showing the chromatin organization of the nucleus and nucleolus of ESCs (open, euchromatic) and differentiated cells (closed, heterochromatic). In ESCs, IGS-rRNA is not processed with consequent lack of mature pRNA [48]. IGS-rRNA impairs the association of TIP5 with TTF1 and TIP5 recruitment to rRNA genes. Consequently, all rRNA genes are kept euchromatic and active in ESCs. Upon differentiation, mature pRNA is produced and promotes TIP5-TTF1 interaction that is productive for TIP5 guiding to rRNA genes and formation of heterochromatin at nucleoli. The formation of silent and heterochromatic rRNA genes coincides with the remodeling of the genome from a euchromatic into a heterochromatic state that favors the exit from pluripotency.

The de novo establishment of silent rRNA genes occurs early in development. In mouse embryonic stem cells (ESCs), the promoter sequences of all rRNA genes lack CpG methylation [47,48] (Figure 2). Hypomethylation of rRNA genes was reported in ground state pluripotent cells, which are cultured in the presence of the 2 inhibitors (2i, MEK inhibitor PD 0325901 and the GSK inhibitor CHIR 99021) and display global DNA hypomethylation [48]. Similarly, rRNA genes lack DNA methylation in developmentally advanced ESCs, which are cultured with serum and the leukemia inhibitory factor (LIF) and have globally high meCpG content [47]. Importantly, de novo establishment of silent rRNA genes occurs shortly after ESC differentiation. Upon exit from the pluripotency state, rRNA genes start to acquire DNA methylation and associate with repressive histone marks such as H3K9me2 and H3K9me3 [48]. Similarly, in plants, all rRNA genes are active in newly germinated seeds, but by 10–14 days after germination and throughout the remainder of vegetative development, 50% of the total rRNA gene pool is selectively silenced [31,59,60]. Thus, the lack of silent rRNA genes is a feature of pluripotency states. Interestingly, several pluripotency factors such as Oct4 and Sox2 were found associated with rRNA genes in both mouse and human ESCs, suggesting further layers of rRNA gene regulation in pluripotency states [61].

In mammalian cells, the factor responsible for the formation and maintenance of silent rRNA genes is the nucleolar remodeling complex NoRC [24,25,43]. NoRC is composed of TIP5 (TTF-I interacting protein 5) and SNF2H, a member of the ISWI subfamily and catalytic subunit of several chromatin-remodeling complexes. NoRC interacts with repressive factors such as DNA methyltransferases [24]. In the past, the energy-dependent nucleolar silencing complex (eNoSC) and the nucleosome remodeling and deacetylase (NuRD) complex have also been implicated in the formation of silent rRNA genes [62,63]. However, both eNoSC and NuRD establish a repressive or poised chromatin state for transcription without affecting DNA methylation, indicating that their role is eventually linked either to the formation of inactive rRNA genes or to the repression of active genes (Figure 1b). Thus, in mammalian cells, NoRC is the only complex that has so far been identified to establish silent rRNA genes. 

The recruitment of NoRC to the promoter of rRNA genes is mediated by the interaction of TIP5 with TTF1 (transcription terminator factor I) and the long non-coding (lnc)RNA promoter RNA (pRNA) [65,66] (Figure 1b). TTF1 is a nucleolar protein that binds to terminator (T) elements, including the T0 sequences at rRNA gene promoter and is implicated in several rRNA regulatory processes, including termination [67,68,69]. pRNA contains rRNA gene main promoter sequences (from ca. −220 to −1 relative to the transcription start site) and originates from the processing of the intergenic spacer rRNA (IGS-rRNA) that in mouse cells is transcribed from the spacer promoter located 2 kb upstream the main rRNA gene promoter [57,66] (Figure 2a). IGS-rRNA is also transcribed by Pol I and in differentiated cells is synthesized from active rRNA genes in the early S-phase, the time when they replicate [57]. Shortly after synthesis, IGS-rRNA is processed into pRNA (ca 200 nt long), a reaction mediated by DHX9 (RNA Helicase A, RHA) [64]. TIP5 is an RNA binding protein and interacts with pRNA through the TAM (TIP5/ARBP/MBD) domain [66,70,71]. pRNA folds into a conserved stem-loop structure that is required for the interaction with TIP5, its recruitment to the rRNA gene promoter and the establishment of silencing [48,72]. Downregulation of pRNA impairs the nucleolar localization of TIP5 and its association with the rRNA gene promoter, causing loss of repressive marks, including DNA methylation [66]. 

There is some confusion in the literature concerning the mechanisms of pRNA-mediated TIP5 recruitment to rRNA genes. A previous report indicated that the 5′-pRNA sequences, corresponding to T0 sequences at the rRNA main gene promoter, can form triple helix with the rDNA promoter [73]. The triple-helix formation involves a double stranded (ds) nucleic acid such as the duplex DNA and a single-stranded (ss) nucleic acid such as RNA [74]. The formation of triplex helices is based on sequence-specific binding rules—the ss nucleic acid binds in the major groove of the targeted ds-DNA through sequence specific recognition of a polypurine-polypyrimidine sequence (Hoogsteen or reverse Hoogsteen base pairing) [75]. Thus, it was a surprising result that pRNA sequences without having this sequence motif could form triple-helix [73]. Although this study did not show any experiment linking pRNA triple-helix and TIP5, for reasons that remain unclear to us, the recruitment of TIP5 to rRNA genes via triple-helix is often reported in many reviews as a model of RNA triple-helix mediated guiding of protein complexes to defined genomic loci [58,76,77,78,79]. However, experiments aimed at testing whether pRNA sequences for triple-helix formation were required to recruit TIP5 to rRNA genes failed to demonstrate the triple-helix recruitment model. Furthermore, this model ignored previous data showing that TTF1, which associates with the rRNA gene promoter in a sequence specific manner, interacts with TIP5 and is required for its recruitment to rRNA genes [43,65]. Recent results, however, proposed a mechanism unifying the role of pRNA and TTF-1 in the recruitment of TIP5 to rRNA genes, showing that the association of TIP5 with TTF-1 tightly depends on the stem-loop structure of pRNA [48] (Figure 2b). Thus, TIP5 recruitment to the rRNA gene promoter likely represents a model of guiding of chromatin regulators to defined genomic sites through the interaction of lncRNAs with sequence specific DNA-binding proteins. 

The recruitment of TIP5 to rRNA genes and consequent formation of silent rRNA genes is developmentally regulated. ESCs lack silent rRNA genes since TIP5 nucleolar localization and recruitment to rRNA genes is impaired [48]. Upon differentiation, TIP5 is guided to rRNA genes and initiates the formation of silent rRNA copies that will be maintained throughout the rest of development. The abrogation of TIP5 binding to rRNA genes in ESCs is mainly due to the impairment of IGS-rRNA processing into the mature pRNA (Figure 2b). While differentiated cells efficiently process IGS-rRNA, this reaction is strongly downregulated in ESCs. Consequently, pRNA is less abundant in ESCs than in differentiated cells such as neural progenitors (NPCs) [48]. Remarkably, introduction of pRNA in ESCs was sufficient to recruit TIP5 to rRNA genes and initiate rRNA gene silencing. Similarly, TIP5 nucleolar localization and interaction with rRNA genes in differentiated cells are abrogated by downregulation of DHX9, a regulator of IGS-rRNA processing [64]. Biochemical analysis determined that the production of mature pRNA is critical for TIP5 recruitment to rRNA genes since mature pRNA promotes the association of TIP5 with TTF1 whereas IGS-rRNA bound to TIP5 abrogates this interaction and impairs TIP5 recruitment to rRNA genes [48]. The critical role of pRNA biogenesis in the establishment of silent rRNA genes represents an important example of how the different features of the same lncRNA can be modulated to regulate chromatin conformation and epigenetic patterns during development. 

Once the silencing of the ribosomal genes has been established, this state is maintained through cell division. In differentiated cells, rRNA genes are replicated in a biphasic manner; active rRNA genes replicate in early S phase whereas silent rRNA genes are duplicated in mid-late S phase [28,54]. Maintenance of the silenced state is also mediated by TIP5 that interacts with silent rRNA genes immediately after their replication [28]. The timing of IGS-rRNA synthesis and processing into pRNA correlate with the inheritance of late-replicating silent rRNA genes mediated by TIP5 and suggest that pRNA-mediated rRNA gene silencing acts in *trans* [57]. A pRNA-mediated mechanism acting in *trans* is also consistent with the results showing that transfection of in vitro synthesized pRNA in ESCs is sufficient to recruit TIP5 to rRNA genes [48]. Thus, the cell carefully tunes the timing of IGS-rRNA transcription/processing so that at the time of the replication of silent rRNA genes novel pRNA moieties are available for TIP5 recruitment and consequent re-establishment of silent and repressive chromatin [57]. TIP5 function can also be regulated through post-translational modification. The acetyltransferase MOF (males absent on the first) acetylates TIP5 at K633 residue and this modification is removed by the NAD^+^-dependent deacetylase SIRT1 (sirtuin-1) [80]. Acetylation of TIP5-K633 impairs the binding of TIP5 with pRNA and consequently the association with rRNA genes. Interestingly, TIP5-K633 acetylation was shown to fluctuate during S phase and, in particular, to increase before the replication of silent rRNA genes, suggesting a mechanism of TIP5 displacement from silent rRNA genes to allow the progression of the DNA replication machinery. 

The de novo establishment or maintenance of silent rRNA genes mediated by TIP5 occurs through several steps. TIP5 associates with DNMTs and, as part of NoRC, remodels the nucleosome located at the mouse rRNA gene promoters through sliding from position −157 to −132 relative to the transcription start site [24,81]. NoRC-mediated nucleosome remodeling was proposed to expose the CpG at position −133 to the action of DNMTs, whose catalytic efficiency is significantly compromised on nucleosome substrate when compared to naked DNA [82,83]. Accordingly, TIP5-mediated repression of rRNA genes depends on the methylation of CpG −133 that is required for transcriptional silencing [23,84]. TIP5 also serves as scaffold for the recruitment of other repressor complexes. TIP5 C-terminal bromodomain recognizes and binds to the H4K16ac at rRNA gene promoter and establishes a repressive chromatin state through the recruitment of SIN3 corepressor complex that includes histone deacetylase 1 (HDAC1) [25,85]. TIP5 also interacts with the histone H3K9 methyltransferase SETDB1 (KMT1E) [86]. Another factor associating with TIP5 is the poly(ADP-ribose)-polymerase-1 (PARP1), an interaction that is mediated by pRNA [87]. PARP1 binds to silent rRNA genes after their replication and its activity is required for the re-establishment and maintenance of silent chromatin during cell division. In plants, the establishment of silent rRNA genes involves similar mechanisms. For example, in *Arabidopsis thaliana*, HDA6 is required for CG and CHG maintenance methylation at silent rRNA genes [31]. 

All these results indicate that a complex regulatory network of lncRNAs and epigenetic and chromatin modifiers are implicated in the establishment and maintenance of silent rRNA genes. Furthermore, these processes are tightly regulated during early development (establishment) and cell division (maintenance). In the next chapters, we will discuss the possible functions of silent rRNA genes in mammalian cells.

## 5. Function of Silent rRNA Genes in Genomic Stability and Genome Architecture

So far, a change in the DNA methylation state of rRNA genes was only reported by comparing pluripotent cells vs. differentiated cells or some cancer types vs. normal/healthy cells. In contrast, to our knowledge, there is no evidence reporting that changes in rRNA transcription in response to cell metabolic or proliferative states or genotoxic stress depend on the number of silent rRNA genes. Thus, rRNA gene activity in cell seems to be modulated independently of the level of methylated rRNA genes. Accordingly, early studies showed that the fraction of nucleosome-packed rRNA genes (silent and inactive copies) present in each differentiated cell does not change even under conditions of high metabolic activities [22]. In line with these results, two yeast strains containing different numbers of rRNA genes (143 and 42 copies) produced the same amount of rRNAs [10]. Interestingly, in the reduced copy strain, the mean number of Pol I loaded on each gene was two-fold higher than in the control strain, suggesting that rRNA synthesis in exponentially growing yeast cells is controlled by the ability of cells to load polymerases and not by the number of active genes. The lack of correlation between rRNA gene copy number and rRNA transcription holds also true for other systems [88,89].

Emerging evidence starts to indicate that the function of silent rRNA genes goes beyond ribosome biogenesis and can play important role in nucleolus structure, genome stability, and architecture. In the following sections we describe the implication of silent rRNA gene chromatin in these processes.

### 5.1. Silent rRNA Genes and Genome Stability 

The highly repetitive nature of rRNA genes makes them particularly sensitive to unscheduled recombination [90]. The mechanisms implicated in the repair of rRNA genes have been extensively described in recent reviews [13,90,91]. Here, we will focus on the role of silent rRNA genes and their chromatin features in safeguarding genome stability. 

In yeast, loss of untranscribed (inactive) rRNA genes makes cells sensitive to DNA damage induced by mutagens due to the high number of heavily transcribed rRNA genes [92]. The results show that the inactive rRNA genes facilitate condensin association and sister-chromatid cohesion, thereby facilitating recombinational repair. The requirement of rRNA genes containing heterochromatic and repressive structures for genome stability is also evident by experiments performed in human cell lines showing that the loss of CpG methylation through inactivation of DNMT1 and DNMT3b or treatment with DNA methylation inhibitors induces the formation of extrachromosomal circular rDNA (ecc), which is an indication of genomic instability [93]. Similarly, studies in *Drosophila* showed that cells lacking H3K9 methylation and RNA interference (RNAi) pathway components display disorganized nucleoli and a substantial increase in ecc repeated rDNAs [94]. This same study determined that heterochromatic structures are important to ensure stability at repetitive sequence, including rRNA genes, by suppressing non-homologous end joining (NHEJ) or other recombination pathways. In differentiated mammalian cells, the impairment of heterochromatin formation at rRNA genes through TIP5 depletion induced genomic instability, including the loss of rRNA genes and centromeric repeats and alterations in the nucleolus structure [54]. Therefore, it appears that silent rRNA copies in mammalian cells do not only protect nucleolar gene stability but also the one of other repeats located outside the nucleolus. Similar observations were reported in another study [95]. Finally, in *Saccharomyces cerevisiae,* mutations in SIR2, a factor regulating silencing, induce an increase in homologous recombination at the rRNA gene locus, which results in the formation of extrachromosomal rDNA circles in the nucleolus that have an impact in cell senescence and aging [96,97]. 

Taken together these results suggest that cells contain multiple copies of rRNA genes not only to produce high rRNA levels but also to preserve genome integrity through the presence of silent heterochromatic rRNA genes.

### 5.2. Function of Silent rRNA Genes and Nucleolus in Genome Organization

#### 5.2.1. Nucleolus in Genome Organization

Increasing evidence indicates that large-scale folding of chromatin may affect gene expression by locating genes to specific subnuclear compartments that allow the concentration of factors (e.g. repressor or activators) and thereby facilitate functions that rely on proteins found in limiting concentrations [98]. Nuclear context is also important for the position of defined genomic region relative to nuclear pores, the nuclear lamina, or the nucleolus [99]. 

In the last decades, several results indicate that the role of nucleolus might go beyond the solely production of ribosomes and it can play important roles in the organization of 3D genome architecture. Clustering of heterochromatin at nucleoli is a phenomenon known to occur in all somatic cells (Figure 2b). For example, centromeres and telomeres often associate to nucleoli [100,101,102]. Similarly, the inactive X chromosome was found to contact the perinucleolar compartment during mid/late-S-phase and it was suggested that this location could be important for faithful duplication of silent chromatin [103]. Recently, MiCEE, a multicomponent ribonucleoprotein complex containing factors of the exosome (C1D, EXOSC10 and EXOSC5), and PRC2 (EZH2, SUZ12, and EED) was found to tether loci of bidirectionally active genes to the perinucleolar region and induce ncRNA degradation and transcriptional silencing by heterochromatin formation [104]. Thus, although rRNA genes in the nucleoli produce the overwhelming majority of RNAs in the cell, the nucleolus is an attractive compartment for repressive genomic domains and, together with the nuclear lamina, represents the hub for the organization of the inactive heterochromatin [105,106,107]. 

In mammalian cells, large portions of the genome associate with the nuclear lamina at the periphery of the nucleus and are identified as lamina-associated domains (LADs). LADs are essentially composed of regions with silent chromatin signatures [107]. Genomic regions positioned in close proximity of the nucleolus are known as nucleolus-associated domains (NADs). Initial genome-wide studies identified NADs using biochemically-purified nucleoli, a method based on the sonication of nuclei, adjusting the power so that nucleoli remain intact while the rest of the nuclei are fragmented [108]. The first two studies mapped the NADs from nucleoli of HeLa, IMR90, and HT1080 human cell lines [109,110]. NADs were found to correspond to regions of low gene densities, low transcriptional levels and repressive histone modifications (H4K20me3, H3K27me3, and H3K9me3). Centromeric and pericentromeric satellite repetitive repeats and subtelomeric regions were also identified as NADs, confirming previous microscopy studies. NADs cover around 40% of the genome and comparison of NADs and LADs revealed substantial overlap [109,111]. Using the same biochemical purification of nucleoli, studies in plant cells (*A. thaliana*) determined that NADs cover around 4.2% of the annotated genome and, excluding rRNA genes, are mainly composed of transposable elements and intergenic regions (both around 35%), followed by low transcribed genes (30%) that display heterochromatic features (CG methylation, H3K9me2, H3K27me1/3), pseudogenes, and tRNAs [112]. 

Interactions of genomic loci with nucleoli have also been identified by measuring the contacts between the rRNA genes and the rest of the genome using Hi-C, a method that serves to study the three-dimensional architecture of genomes [113]. Recent analyses using Hi-C data sets from lymphoblastoid (LCL) and erythroleukemia (K562) cells revealed that rRNA genes contacts are enriched in segments of closed, repressed, and late replicating chromatin, as well as CTCF binding sites [114,115]. Interestingly, developmentally regulated *Hox* genes were found rarely localized in proximity to the rRNA gene arrays. Analysis of Hi-C data from IMR90 cells [115] revealed that 74% of NADs reside in B2/B3-type constitutive heterochromatic chromosomal regions [111]. Circularized chromosome conformation capture sequencing (4C-seq) has also been used to identify contacts between rRNA genes and the rest of the genome. In HEK293T cells, the intergenic region of rRNA genes was shown to associate with pericentromeric and centromeric repetitive sequences, a result consistent with previous observations [116]. More recently, the 4C-seq method was employed to identify genomic contacts with rRNA genes using an Eμ-Myc mouse model of spontaneous MYC-driven B cell lymphoma [117]. A recently developed method, split-pool recognition of interactions by tag extension (SPRITE), enabled genome-wide detection of higher-order interactions within the nucleus [99]. This method can simultaneously measure RNA and DNA interactions and allowed the identification of genomic regions contacting rRNA transcripts that correspond to DNA located around the nucleolus. This analysis revealed that regions that are linearly close to the centromere are closer to the nucleolus, a result that is consistent with previous observations that centromeres often co-localize on the periphery of the nucleolus. Interestingly, actively transcribed regions are excluded from the nucleolar compartment even when they reside in linear proximity to a centromere, supporting the idea that the positioning close to nucleolus is mainly linked to repressive states. This analysis also provided an important distinction between NADs and LADs by determining the presence of inter-chromosomal contacts at the same nucleolus whereas lamina-associated interactions generally occur between regions that are linearly close to each other rather than between chromosomes [99].

The finding that a large portion of NADs corresponds to LADs led to suggest that the nucleolus and nuclear lamina could serve as interchangeable scaffolds for the localization of heterochromatic domains. Accordingly, evidence indicates that LADs from the mother cell after completion of cell division can be positioned to the nucleoli of the daughter cells [118,119]. Similarly, NADs can relocate from nucleoli in close proximity of the nuclear envelope after mitosis [109]. Interestingly, disruption of nucleoli by inhibition of rRNA gene transcription with Actinomycin D was reported to increase the relocation of repressed and late replicating loci usually placed at the nucleolar periphery [119], suggesting a role of the nucleolus as compartment for the re-establishment of repressive domains after replication. An involvement of the nucleolus in the spatial organization of chromosomes as well as telomere maintenance also comes from study in plants. In *A. thaliana nuc1* (Nucleolin) null mutants, which show altered rRNA gene expression and nucleolar structure, NAD composition changes, telomere association with the nucleolus is decreased, and telomeres become shorter [112].

#### 5.2.2. Function of rRNA Gene Chromatin in the Genome Organization in Disease and Development

The nucleolus is a membrane-less nuclear compartment that appears to assemble through phase separation of their molecular components [4]. The formation of the nucleolus depends on active rRNA transcription and pre-rRNA is the key player in seeding nucleolus formation [120]. A well-described phenomenon is the nucleolar segregation caused by inhibition of rRNA gene transcription with the formation of “nucleolar caps” around the nucleolar remnant [121]. Thus, the morphology and size of the nucleolus is linked to transcriptional activity, which in turn depends on cell growth and metabolism and also developmental state. Consequently, rRNA gene transcription state can influence the nucleolar structure and in turn genome structure, including the organization of NADs. 

Structural changes at the nucleolus are often observed in cancer [6]. A link between rRNA gene chromatin state and genome organization in cancer has recently been highlighted in lymphomagenesis [117]. During the progression from premalignancy to malignancy, UBF associates with a fraction of inactive genes and remodels their chromatin into an active state. This process does not include the reactivation of silent methylated rRNA genes. 4C-seq analyses identified a subclass of NADs that interacts with rRNA genes. Concomitant with the activation of rRNA genes, the composition of these NADs changes. Some of these NADs show reduced expression in malignant cells and are enriched for genes involved in B-cell differentiation, a pathway that is often compromised in hematologic malignancies [122]. Remarkably, some of the rRNA genes-NAD interactions that change during malignant progression require the active chromatin state of the rRNA genes but not active transcription, supporting a role of the chromatin structure of rRNA genes in shaping genome organization.

A link between the chromatin and transcriptional state of rRNA genes and genome organization holds also true in ESCs, where all rRNA genes are active due to the lack of DNA methylation and repressive histone marks such as H3K9me2 and H3K9me3 [47,48]. Acquisition of heterochromatic and silent rRNA genes occurs only upon exit from pluripotency and initiation of differentiation (Figure 2b). The euchromatic state of rRNA genes in ESCs resembles the structure of the rest of the ESC genome that is generally less condensed and largely devoid of compact heterochromatin blocks compared to differentiated cells [123,124]. This open and transcriptionally permissive state of ESC chromatin well reflects the plasticity of ESC genome that must have the ability to enter any distinct transcriptional programs for lineage specification [125,126]. Accordingly, upon differentiation, large-scale genome silencing takes place and the open ESC chromatin undergoes structural remodeling toward a highly condensed heterochromatic and transcriptionally repressed form, including clustering of heterochromatin at the nucleolus or at the nuclear periphery [48,127,128,129,130] (Figure 2b). The de novo establishment of rRNA gene silencing upon ESC differentiation coincides with the downregulation of rRNA gene transcription, indicating that the reduction in nucleolar transcription is an early event during the differentiation of pluripotent stem cells [48,131,132]. Accordingly, stable expression of fibrillarin, a specific marker for the dense fibrillar component and indispensable for ribosome biogenesis, prolongs the pluripotent state of mouse ESCs cultured in the absence of leukemia inhibitory factor (LIF). Similarly, partial knockdown of fibrillarin and treatment with Actinomycin D induce the expression of differentiation markers in the presence of LIF and promote stem cell differentiation into neuronal lineages [132]. Differentiation of human ESCs driven by Activin A caused reduction of rRNA synthesis and UBF displacement in only 6 h, while the activation of germ layers specific transcriptional programs happens within 48 h [131]. In *Drosophila*, disruption of components of Pol I regulatory complex composed of Under-developed (Udd) and TAF1B reduced ovarian germline stem cell (GSC) proliferation whereas the increase of Pol I transcription delayed differentiation [133]. These results suggest that the regulation of rRNA gene transcription in ESCs is a critical aspect of cell fate determination and that the reduction of rRNA synthesis is required for ESC differentiation. Due to their highly proliferative state, it is likely that ESCs have a high demand for ribosome synthesis and reduction of rRNA transcription might affect the retention of self-renewal capability. Surprisingly, however, the impairment of heterochromatin formation at rRNA genes during ESC differentiation abolishes the exit from pluripotency, suggesting a role of silent rRNA genes in cell fate specification that might go beyond the control of ribosome biogenesis [64]. Accordingly, targeting of heterochromatin in the nucleolus of ESCs through the addition of mature pRNA induced the remodeling of the open and euchromatic ESC genome into a condensed heterochromatic form. These changes included the appearance of highly condensed heterochromatic blocks outside the nucleolus, a structure resembling the genome organization found in differentiated cells [48]. These ESCs containing a heterochromatic nucleolus and nuclear genome display a global increase in the repressive histone mark H3K9me2, increased expression of genes involved in cell differentiation and developmental processes, and loss of pluripotency due the inability to form teratoma. Thus, the formation of heterochromatin in the nucleolus at rRNA genes promotes heterochromatinization of the rest of the nuclear genome, and this process is required to exit from pluripotency. The link between rRNA genes and the chromatin architecture of the rest of the genome is also supported by previous results in *Drosophila* showing that the deletion of rRNA repeats in the Y chromosome reduces heterochromatin content elsewhere in the genome in much the same manner as mutations in known protein heterochromatin components [134]. Induced deletions of rRNA genes affect the expression of hundreds to thousands of euchromatic genes throughout the genome of males and females and these affected genes significantly overlap with genes affected by natural polymorphisms on Y chromosomes [135]. A similar observation was also found in mouse embryo fibroblast cells NIH3T3, where half of rRNA genes are heterochromatic. Knockdown of TIP5 induced not only a decrease of rRNA gene silencing but also the reduction of silent histone marks at pericentric heterochromatin [54,95]. 

Together, all these results indicated that the nucleolus is not only the nuclear compartment where ribosomes are produced but it is also a central component of nuclear architecture that models the genome according to cell state. Likewise, the function of rRNA genes might not only be limited to the synthesis of rRNA but might play a role in the organization of nuclear genome architecture.

## 6. Conclusions

In this review we summarized increasing evidence of the role of the nucleolus in genome organization and cell fate. We described the distinct rRNA gene classes and their role in nucleolar transcription with a special focus on the mechanisms regulating the formation of silent rRNA repeats and their impact in genome stability and architecture. We described that rRNA gene silencing is regulated during development and linked to the organization of the genome outside the nucleolus. Emerging evidence showed that the nucleolus, together with the nuclear lamina, represents the hub for the organization of the inactive heterochromatin. Changes in the rRNA transcription and chromatin states observed during development or disease can affect nucleolus structure and, consequently, influence the 3D architecture of the genome. Although the mechanisms remain yet elusive, an attractive hypothesis is that the alteration of the chromatin state at rRNA repeats alters the nucleolus in its structure and protein composition, allowing the concentration of factors required for the establishment of repressive states. Until now, however, the major limitation to study the role of the nucleolus in genome organization has been the difficulty to precisely map chromatin domains associated with the nucleolus (NADs) at genome-wide level as done for LADs [136]. Indeed, the use of the DamID method was extremely successful to dissect LAD composition and function even in single cell, providing basic principles of genome organization during development and disease states [137,138]. In contrast, the full understanding of how the nucleolus is linked to nuclear genome organization has been hampered by the fact that the nucleolus is a membraneless compartment, limiting the application of the successful DamID technology. Mapping of genome-nuclear lamina interactions by DamID has estimated that ∼35% of the mammalian genome can interact with the NL in any tested cell type, indicating that only a fraction of the genome in a single cell can interact with the nuclear lamina. This hypothesis was recently confirmed by single cell analysis and supported by the observation that after mitosis only a fraction of LADs return to the nuclear periphery in the daughter cells, while many others associate with nucleoli [118,137]. Recent advances in imaging and genomic technologies can now allow a better understanding of the crosstalk between nucleolar and nuclear chromatin and provide a better view of genome compartmentalization in the cell nucleus that until now was mostly limited to domains associated with the nuclear lamina. The establishment of novel methods to map at high-resolution genomic contacts with the nucleolus and their application in single cell analysis will allow defining nucleolar dynamics and determining the molecular mechanisms of how the organization of the genome around the nucleolus is established, its functional role, and its dynamics during development and disease.
